# Nutritional, Therapeutic, and Functional Food Perspectives of Kale (*Brassica oleracea* var. *acephala*): An Integrative Review

**DOI:** 10.3390/molecules30214214

**Published:** 2025-10-28

**Authors:** Aleksandra Łukaszyk, Inga Kwiecień, Anita Kanik, Eliza Blicharska, Małgorzata Tatarczak-Michalewska, Wojciech Białowąs, Katarzyna Czarnek, Agnieszka Szopa

**Affiliations:** 1 Department of Medicinal Plant and Mushroom Biotechnology, Faculty of Pharmacy, Jagiellonian University, 9 Medyczna St., 30-688 Kraków, Poland; aleksandra.lukaszyk@student.uj.edu.pl (A.Ł.); inga.kwiecien@uj.edu.pl (I.K.);; 2 Department of Pathobiochemistry and Interdisciplinary Applications of Ion Chromatography, Medical University of Lublin, 1 Chodźki St., 20-093 Lublin, Poland; elizablicharska@umlub.pl (E.B.); malgorzata.tatarczak-michalewska@umlub.pl (M.T.-M.); 3 Doctoral School, Catholic University in Lublin, Konstantynów 1F St., 20-708 Lublin, Poland; 4 Institute of Medical Science, Faculty of Medical Sciences, The John Paul II Catholic University of Lublin, 1H Konstantynów St., 20-708 Lublin, Poland; katarzyna.czarnek@kul.pl

**Keywords:** *Brassica oleracea* var. *acephala*, kale, glucosinolates, functional food, antioxidant activity, chemoprevention, phytochemicals, anti-inflammatory compounds, nutritional value

## Abstract

Kale (*Brassica oleracea* var. *acephala*) is a non-heading leafy vegetable of the *Brassicaceae* family, widely recognized for its dense nutritional profile and diverse phytochemical composition. This review provides a comprehensive and up-to-date synthesis of kale’s botanical characteristics, cultivation practices, chemical constituents, biological activities, and applications in pharmacy, functional foods, and cosmetics. Importantly, this work highlights the novelty of kale’s multifunctional role. Kale is particularly rich in vitamins (A, C, K), minerals (Ca, Fe, K), dietary fiber, glucosinolates, polyphenols, carotenoids, flavonoids, and chlorophylls, which contribute to its classification as a “superfood.” In this article the discussion of the health-promoting effects of glucosinolates and their enzymatic degradation products, such as isothiocyanates, indoles, and nitriles, highlighting their antioxidant, anti-inflammatory, anticancer, antimicrobial, and lipid-lowering properties, was performed. Moreover, key compounds including sulforaphane, indole-3-carbinol (I3C), and diindolylmethane (DIM) are emphasized for their roles in chemoprevention, hormonal regulation, and cellular protection. The review also summarizes recent in vivo and clinical studies demonstrating kale’s potential in reducing the risk of chronic diseases such as cardiovascular disorders, type 2 diabetes, and hormone-related cancers. The effects of kale on the composition of the gut microbiome, glycemic control, and cholesterol metabolism are also discussed. Advances in plant biotechnology, including micropropagation, somatic embryogenesis, and metabolite enhancement, are also discussed. Overall, this review supports the integration of kale into health-oriented dietary strategies and highlights its relevance in preventive medicine, food innovation, and cosmeceutical development.

## 1. Introduction

The Brassicaceae (Cruciferae) family consists of 349 genera and about 4000 species [1]. With the discovery of new species and the use of modern techniques that allow for a more detailed study of their genetic diversity, the number of species is constantly changing [2]. Species of the *Brassicaceae* family are known as a very diverse group of plants due to their ability to spontaneously crossbreed [3]. One of the main species of the *Brassicaceae* family with valuable health-promoting properties is *Brassica oleracea* var. *acephala* DC. (kale, green kale) [4]. A diet rich in kale may help reduce the risk of chronic diseases and some types of cancer, such as prostate, colon, bladder, pancreas, stomach, thyroid, lung, and skin cancer. It is believed that the presence of phytochemicals such as glucosinolates (GLS), polyphenols, and carotenoids may be responsible for its health-promoting activity [2,5].

Glucosinolates are characteristic compounds for plants of the *Brassicaceae* family; they are composed of a β-D-thioglucose group connected by a sulfur atom to the aglycone. As a result of cell damage, enzymatic hydrolysis of glucosinolates occurs by myrosinase (thioglucoside glucohydrolase; E.C. 3.2.1.147) or beta-thioglucosidase. In this process, unstable oximes are released, from which stable and unstable isothiocyanates (ITC) are formed [6,7]. Indoles are formed by hydrolysis of GLS. The available data indicates that the total number of characterized GSLs is estimated to fall within the range of 88 to 137 [8]. They can be divided into three classes: aliphatic, benzenic, and indole [9].

Kale is one of the oldest varieties of the *Brassicaceae* family and has been considered a food plant since about 2000 BC [10]. *B. oleracea* var. *acephala* comes from wild cabbage and is a subvariety of the headless cabbage [11].

*B. oleracea* var. *acephala* is known in English-speaking countries as “kale” and in Brazil as “couve” [12]. In the Southern African sub-region, it is commonly cultivated commercially and domestically under the name of “Rugare” or “Covo” in Zimbabwe, “Sukuma wiki” in East Africa, and “muRhodesia” in northern South Africa. *B. oleracea* var. *acephala* is also called “walking stick cabbage” due to the tall woody stem the plant produces as it grows upward for many months [13,14,15].

The aim of this review is to provide an up-to-date, integrative analysis of the morphological characteristics, phytochemical composition, nutritional value, biological activity, and functional food potential of kale. Furthermore, this paper highlights the latest advances in biotechnological approaches for the cultivation and enhancement of kale’s bioactive properties, supporting its relevance in modern nutrition science and preventive healthcare.

This review provides a comprehensive and up-to-date synthesis of *B. oleracea* var. *acephala* research, integrating recent findings in pharmacognosy, phytochemistry, plant biotechnology, pharmacology, phytotherapy, functional food development, and cosmeceutical applications. Unlike previous reviews, it emphasizes kale’s relevance in modern preventive medicine and cosmetic science, as well as plant biotechnology. Topics such as industrial-scale processing and economic aspects of cultivation are beyond the scope of this manuscript.

## 2. Morphological Appearance and Varieties

**Figure 1:**
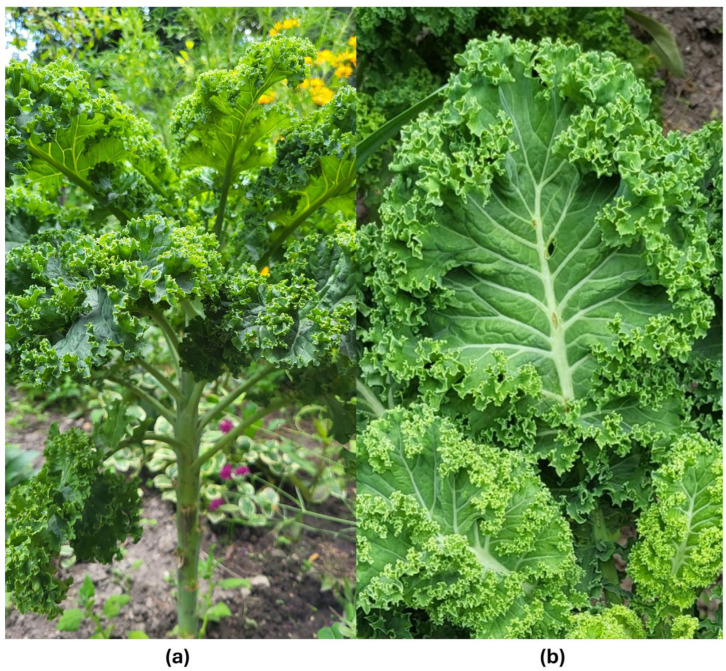
*Brassica oleracea* var. *acephala*; (**a**) morphological appearance of a plant, (**b**) morphological appearance of the leaf (photo by Agnieszka Szopa).

Kale is a biennial, non-heading vegetable whose edible part is long, pinnate, curled leaves of oval or oblong-oval shape. Leaves of *B. oleracea* var. *acephala* are green in color and have a jagged structure. (Figure 1). During the long growing season, the central stem can grow to 60 cm or more. In the second year of growth, it produces yellow flowers with four petals, arranged loosely in clusters. The fruits are dry capsules called siliques [16].

Kale can be divided into at least three groups based on origin: curly kale, collard kale, and Italian kale. Curly kale (Scottish type, *Brassica oleracea* convar. *acephala* var. *sabellica*) is mainly grown in Northern Europe and is considered an example of an ideal kale. Italian kale (Italian variety, Lacinato type, *Brassica oleracea* convar. *acephala* var. *palmifolia*) was originally cultivated in Tuscany and is characterized by dark green leaves with a wavy edge. Collard (*Brassica oleracea* convar. *acephala* var. *viridis*) occurs mainly in the southern United States and is characterized by large, flat, round leaves that resemble wild cabbage. Russian kale (Russian kale, Siberian type, *Brassica napus* var. *pabularia*) can also be distinguished. Despite the wide distribution of plants and their morphological diversity of kale, there has not been much scientific research on this plant [17]. The name ‘kale’ usually refers to the species *B. oleracea* var. *acephala*, but it also includes other market classes such as *B. napus* var. *pabularis* (Russian-Siberian kale), *B. oleracea* var. *alboglabra* (Chinese kale), and some varieties of *B. oleracea* var. *viridis* (collard greens) [18]. The division of kale varieties with examples and morphological features is presented in Table 1.

Moreover, *Brassica oleracea* var. *acephala* is also referred to as ornamental kale. In this sense, it is a leafy plant valued for its decorative qualities. It takes on colors from intense white to shades of red, pink, and lavender blue. Its spectacular leaves and resistance to low temperatures make it an extremely valued decorative element in gardens and landscape arrangements [19].

High morphological variability in kale can occur both within populations, due to cross-breeding, and between populations, due to intensive selection by farmers and adaptation to changing environmental conditions [3]. Kale varieties differ in leaf color and degree of curliness, stem height, and resistance to low temperatures. There are low (up to 20 cm), medium-high (20–80 cm), and high (over 80 cm) varieties. The temperature up to −15 °C is well tolerated by the varieties: low Kapral and high Kadet, which have strongly curly, dark green leaves [20].

## 3. Occurrence and Cultivation

*B. oleracea* var. *acephala* originates from the eastern Mediterranean area [21]. Kale is widely cultivated in central and northern Europe and North America. It is grown in the Black Sea area and in the central and northern parts of the Iberian Peninsula. It is better adapted to the conditions there than the more sensitive cauliflower or broccoli [22,23].

For optimal cultivation, kale should be grown in rotation with non-*Brassicaceae* species and returned to the same soil only every 3–4 years. It thrives best in clay soils with a neutral to slightly acidic pH. Due to its high nutrient demand, soil preparation should include thorough digging and the incorporation of organic fertilizer. Adding nitrogen fertilizer has a positive effect on kale crops by improving vegetative growth and delaying premature flowering. However, this is associated with the risk of nitrate accumulation in plant tissues and contributes to environmental pollution [2].

For the fall and winter seasons, kale should be sown at room temperature about 12–14 weeks before the first frost and transplanted into the field after 4 weeks. In spring, it should be sown 4–6 weeks before the last frost and transplanted into the field after the last frost. The seeds germinate in 4–7 days [18].

The leaves can be harvested 4–6 weeks after planting, depending on the expected size and tenderness of the leaf. Harvesting takes place in late autumn and lasts all winter. Kale is harvested several times by tearing off older leaves on the stem, which stimulates the plant to produce new leaves and increases yields. Another practice involves completely removing the young plant. Due to the perishability of kale leaves after harvesting, they should be preserved or used for consumption as soon as possible. In conditions of high air humidity (over 95%) and a temperature of 0 °C, they can be stored for 4 weeks [2,24].

*B. oleracea* var. *acephala* tolerates low temperatures and frost well [25]. It can therefore overwinter in the ground and be harvested until spring. It is also drought-resistant but requires increased water content, which is why it prefers moist air and soil [24]. The popularity of kale may be related to its high tolerance for unfavorable environmental conditions, which makes it adapt well to climate change [25]. French varieties are resistant to *Plasmodiophora* root disease in *Brassicaceae*, and Portuguese varieties are resistant to *Peronospora parasitism* [22]. Farmers harvest the younger, tender leaves for human consumption and the older ones as animal feed [21].

In research conducted by Ku and Juvik [26], adding methyl jasmonate (MeJa) to kale before harvest to mimic biotic stress has been shown to increase antioxidant activity and total phenolics in plant tissue. MeJa can be applied before harvest, as it has been approved as a safe compound for use for this purpose [27].

## 4. Chemical Composition

The United States Department of Agriculture (USDA) plant commodities database indicates that kale is a very good source of many health-promoting nutrients (Table 2) [7,28,29]. Among the vegetables of the *Brassicaceae* family, *B. oleracea* var. *acephala* has the highest content of minerals such as calcium, potassium, and iron, and vitamins such as vitamins A, B_1_, B_2_, E, C, and PP [30]. The calcium found in kale is characterized by high bioavailability. The vitamin C content in *B. oleracea* var. *acephala* is approximately 110 mg/100 g of fresh weight and depends on the maturity of the raw material, storage conditions, and plant growth. Studies on the content of various components in kale have shown that the content of vitamin C increases with the growth of the plant. In turn, the content of minerals remains constant during the growth of the plant [2,31]. *B. oleracea* var. *acephala* is second only to Brussels sprouts among the cruciferous vegetables with the highest protein content. Kale contains about 3 mg of amino acids per 100 g of fresh leaves. As many as 1/3 of kale’s amino acids are exogenous amino acids, such as lysine and leucine [32].

**Figure 2:**
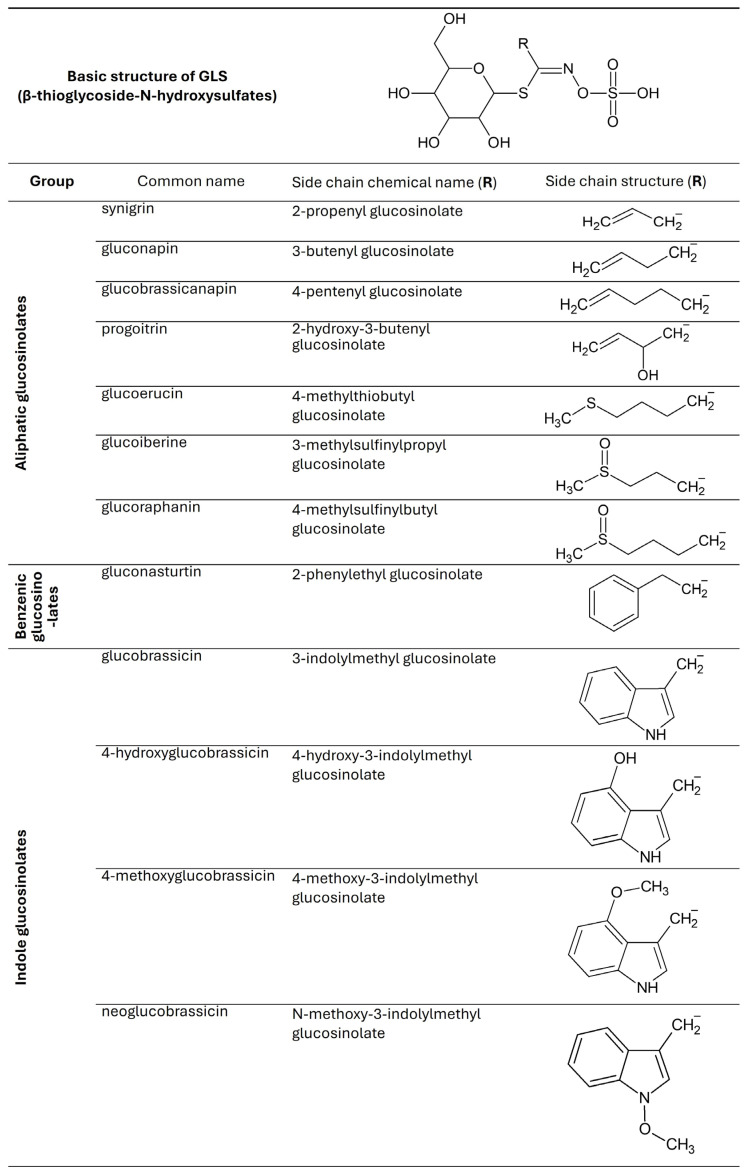
Chemical structures of glucosinolates found in *Brassica oleracea* var. *acephala*.

Fresh kale contains from 2.25 to 93.90 μmol/g dry weight (DW) glucosinolates, but their concentration varies depending on the region of the world from which it comes. The GLS content in vegetables depends on temperature, the presence of pathogens, soil fertility, and the amount of sulfur, nitrogen, and water in the substrate [33]. Studies show that GLS production in kale is induced by damage and wounds, which can be caused by diseases and insects. Figure 2 shows the GLS found in kale [2,7,34].

**Figure 3:**
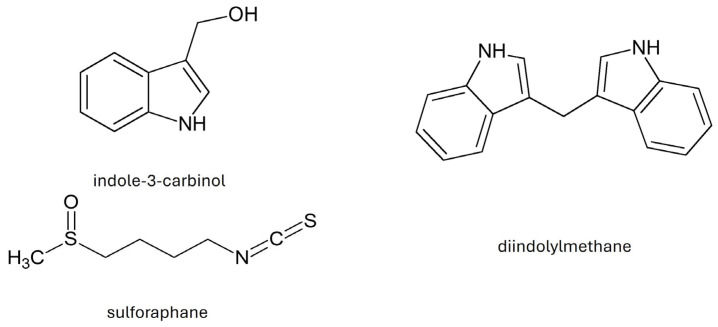
Chemical structure of selected indoles and sulforaphane.

As a result of the decomposition of glucosinolates, their active metabolites—isothiocyanates—are formed (Table 3) [7,34]. As a result of the decomposition of glucobrassicin, the most valuable product is formed, i.e., indole-3-carbinol (I3C). In the process of I3C condensation, diindolylmethane (DIM) [33] is formed. *B. oleracea* var. *acephala* also contains sulforaphane (4-methylsulfinylbutyl isothiocyanate), which belongs to the isothiocyanates (Figure 3) [32].

It has been shown that in 10-day-old kale seedlings, the dominant GLS are glucobrassicin and sinigrin, with researched health benefits. Kale contains less glucobrassicin compared to other *Brassica* vegetables, such as Brussels sprouts or broccoli [2]. Fresh kale vegetables have the most GLS, and their amount decreases with storage time. Prolonged storage, particularly under suboptimal conditions, can lead to the degradation of GLS [33].

In a study by Korus et al. (2014) [35], fresh kale leaves contained 26.87 μmol of total GLS per gram of dry weight (DW). After 12 months of storage, the total GLS content decreased significantly depending on the preservation method. In frozen products, the average GLS concentration was 15.59 μmol/g DW; in canned products, it was 10.73 μmol/g DM; and in dried products, the GLS content ranged from 9.09 to 13.81 μmol/g DW, depending on the drying technique (e.g., hot air drying versus freeze-drying) [35].

**Figure 4:**
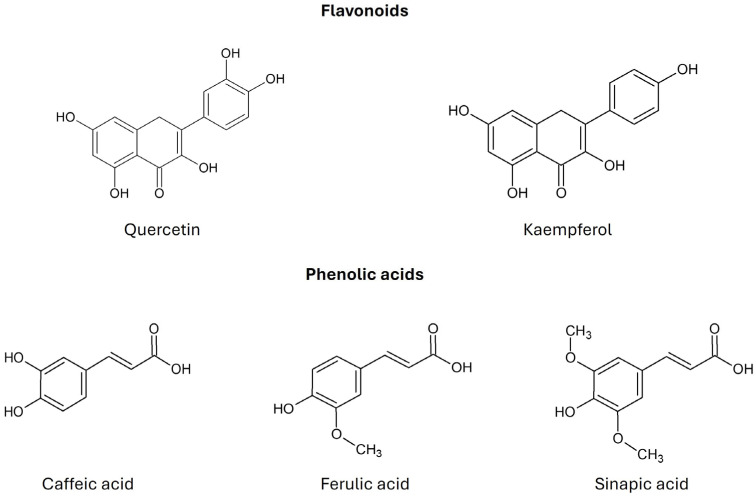
Chemical structure of flavonoids and phenolic acids found in *Brassica oleracea* var. *acephala* (according to Šamec et al., 2019 [2]).

Kale is characterized by a significant content of β-carotene, α-carotene, lutein, and zeaxanthin, the color of which (from yellow to red) is masked by a large amount of chlorophyll (green color). Kale has a higher content of β-carotene than broccoli, cabbage, cauliflower, or Brussels sprouts [7]. Sikora and Bodziarczyk (2012) [36] reported an average β-carotene content of 6.40 mg/100 g fresh weight in raw kale leaves (cv. Winterbor). Lutein and zeaxanthin are stereoisomers that commonly co-occur in nature [36]. According to Holden et al. (1999), kale contains approximately 40 mg of lutein + zeaxanthin per 100 g of fresh weight [37]. Kale is considered one of the richest dietary sources of lutein, surpassing most other orange- or yellow-colored fruits and vegetables [30]. Kale also contains many flavonoid compounds, such as quercetin, kaempferol, and their derivatives. Phenolic acids include caffeic acid, ferulic acid, and sinapic acid (Figure 4) [2]. Anthocyanins have been identified in the red variety of kale in the form of cyanidin glycosides [7].

## 5. Biological Activity and Pro-Health Importance

Kale ranks 15th on the list of so-called “superfoods” (out of 47 vegetables and fruits tested) [38]. Superfoods are also known as functional foods, meaning they can reduce the risk of certain diseases or be used to treat certain conditions. Their use is expected to help lower healthcare costs for an aging population. Undoubtedly, the health-promoting ingredients found in kale are GLS, carotenoids, and flavonoids, as well as vitamins and minerals [2,17,18].

A significant portion of the compounds found in kale exhibit antioxidant activity [7]. They protect the body’s cells from the effects of free radicals, which can damage cellular structures and lead to cancerous transformation [24].

Antitumor activity of *B. oleracea* var. *acephala* is also associated with the composition and presence of compounds that can block the disease at many stages. Healthy people can consume kale as part of primary chemoprevention, the aim of which is to prevent the development of neoplastic changes. These include mechanisms such as the induction of enzymes involved in detoxification or the initiation of DNA repair mechanisms. When carcinogenesis occurs, kale can be used in secondary chemoprevention, which consists of reversing the neoplastic changes that have occurred. This action is associated with the induction of apoptosis, inhibition of angiogenesis, and inhibition of cancer cell proliferation [33]. According to Ortega-Hernández et al., the consumption of *B. oleracea* var. *acephala* by patients undergoing chemotherapy is suggested as a chemopreventive measure, which involves supporting the therapy and maintaining its effects thanks to the presence of glucosinolates and related isothiocyanates [7].

Isothiocyanates (sulforaphane) and indoles such as I3C and DIM have the strongest anticancer properties. The mechanism of anticancer action of glucosinolates is based on the induction of the expression of phase I and II enzymes, which participate in the metabolic activation of carcinogens, inhibition of DNA-damaging factors, participation in the metabolism of estrogens, and limitation of the transformation of initiated cells and restoration of their ability to undergo apoptosis [39]. Listed in Table 3, isothiocyanates reduce the risk of cancer, diabetes, atherosclerosis, and cardiovascular diseases [40]. Sulforaphane, I3C, and 3-methyl-sulfinylpropyl isothiocyanate may reduce the risk of estrogen-dependent cancer, prostate, liver, colon, pancreas, and melanoma [7]. I3C induces the formation of 2-α-hydroxyestrone, a factor that maintains normal breast tissue and reduces the risk of breast cancer [41]. Sulforaphane protects nerve cells from oxidative damage in a mouse model of Parkinson’s disease and participates in the elimination of *Helicobacter pylori* [42,43] (Table 4).

Sinapic acid inhibits NF-κB, which regulates inflammation and plays a role in the response to infections. It also has antiproliferative effects on breast cancer cell lines. Ferulic acid has antioxidant, anti-inflammatory, antidiabetic, antihypertensive, antimicrobial, and antiviral effects [7]. Flavonoids such as quercetin and kaempferol have antioxidant, anti-inflammatory, antimutagenic, anti-edematous, antiallergic, antiatherosclerotic, gastroprotective, neuroprotective, normotensive, vasodilatory, and antiproliferative effects [7,12].

β-Carotene, zeaxanthin, and lutein present in kale are converted in the body into vitamin A, which is essential for the proper functioning of vision processes. Vitamin A prevents the occurrence of problems with vision in low light, more widely known as night blindness. Lutein and zeaxanthin protect the retina from the effects of free radicals because they are components of the macula [46]. β-Carotene and lutein have antioxidant effects, participate in the protection of the skin from UV rays, stimulate the immune system, regulate the cell cycle and growth factors, and modulate intercellular signaling pathways. Thanks to their antioxidant and immunomodulatory effects, they can protect against cardiovascular diseases, cataracts, diabetes, prostate cancer, and cancers in the digestive tract. Lutein has antiproliferative, neuroprotective, antidiabetic, and apoptosis-inducing effects [7].

Chlorophyll a and b participate in the detoxification of the body through strong connections with harmful compounds, thanks to which less of them reach the body’s tissues [47]. Vitamin E is involved in stabilizing cell membranes and prevents oxidative damage to tissues [2]. The beneficial health effects of the ingredients contained in kale leaves are presented below (Table 5).

## 6. Overview of the Newest Scientific Literature

### 6.1. Antioxidant Potential

In the study conducted by Aydin, it was found that ethanol extract from the flowers of B. oleracea var. acephala showed higher total antioxidant activity than ethanol extract from the stems of this plant. The results were expressed in µg ascorbic acid equivalent AAE/mL and amounted to 146.56 µg AAE/mL and 80.35 µg AAE/mL, respectively. The same study showed that ethanol extracts from the flowers of *B. oleracea* var. *acephala* have higher 2,2-diphenyl-1-picrylhydrazyl (DPPH) radical scavenging activity (80.80% inhibition) than ethanol extracts of this plant from stems (13.25% inhibition). As the extract concentration increased, the antioxidant activity increased [49].

In an experiment conducted by Çömlekçioğlu et al. [50], the seasonal variability (from the end of November to the end of March) of the antioxidant activity of the methanol extract from the leaves of *B. oleracea* var. *acephala* was examined. From January, when the temperature began to rise, the DPPH radical scavenging capacity increased and ranged from IC_50_ = 1.91 to 1.41 mg/g DW. In turn, using the ferric reducing antioxidant power (FRAP) method, the values ranged from 29.77 µg AAE/g to 13.43 µg AAE/g [50].

Lučić et al. revealed in their research using the FRAP method that the antioxidant activity of methanol extracts from the leaves of *B. oleracea* var. *acephala* was 29.35 µmol Fe^2+^/g dry weight (DW) [24].

In the study conducted by Băbeanu et al. [23], for the extraction of antioxidant enzymes, fresh tissue was homogenized with 0.1 M phosphate buffer (pH 7.5) containing 0.1 mM ethylenediaminetetraacetic acid (EDTA), and the antioxidant activity of an 80% aqueous:methanol (1:10 *w*/*v*) of *B. oleracea* var. *acephala* leaf extract was proven by measuring the enzymatic activity of superoxide dismutase (SOD), catalase (CAT), and soluble peroxidase (POX). These values were IC 50% = 30.06 mg, 38.6 mM H_2_O_2_/min/g of fresh mass, and 50.33 ΔA/min/g of fresh mass, respectively [23].

### 6.2. Antimicrobial Activity

The study conducted by Aydin reported that ethanol extract from the flowers and stem of *B. oleracea* var. *acephala* exhibited antibacterial activity against *Bacillus subtilis*, *Enterobacter aerogenes*, *Enterococcus faecalis*, *Gordonia rubripertincta*, *Klebsiella pneumoniae*, *Proteus vulgaris*, and *Salmonella enterica*. The Minimum Inhibition Concentration (MIC) values of the tested extracts ranged from 0.625 mg/mL to 5 mg/mL [49].

Another study conducted by Gediz Ertürk et al. documented the antibacterial activity of ethanol extract from the leaves of *B. oleracea* var. *acephala* against the Gram-positive bacteria *Bacillus subtilis*, *Clostridium perfringens*, *Listeria monocytogenes*, *Staphylococcus aureus*, and the Gram-negative bacteria *Escherichia coli*, *Proteus vulgaris*, *Pseudomonas aeruginosa*, and *Salmonella enterica*. There is no data describing the MIC values in the article [51].

In research conducted by Aydin, the antifungal effect of the ethanol extract from the flowers and stem of *B. oleracea* var. *acephala* against *Candida albicans*, *Candida parapsilosis*, *Candida tropicalis*, and *Saccharomyces cerevisiae* was confirmed. MIC values ranged from 0.625 mg/mL to 1.25 mg/mL [49].

According to Gediz Ertürk et al., kale leaf extract showed antifungal activity against *Candida albicans* and *Aspergillus niger* [51].

### 6.3. Anti-Inflammatory Effect

A study conducted by Raychaudhuri et al. [52] showed the anti-inflammatory effect of *B. oleracea* var. *acephala* administered in a high-fat (45% fat) (HF) diet to mice. Mice were given an HF diet or an HF diet supplemented with 4.5% powdered dried *B. oleracea* var. *acephala* (0.12 g per 30 g mouse/day) for 2 weeks. At the end of the 2nd week, the experimental groups received water with the addition of 3% dextran sodium sulfate (DSS) to induce inflammation for a week, while the control group received water alone. It was proven that food with the addition of *B. oleracea* var. *acephala* effectively reduced the expression of IL-6, IL-1b, TNFα, NfKB, and iNOS, as well as the F4/80 gene and protein, which had been previously increased by the supply of DDS. Their levels did not differ compared to the levels of these genes in the control group [52].

The anti-inflammatory effect was confirmed in another study conducted by the same researchers on mice fed an HF diet enriched with kale. In this test, mice were given 0.23 g/30 g body weight of dried and powdered leaves of *B. oleracea* var. *acephala* daily for 12 weeks. This diet significantly reduced the expression of MCP-1 in serum while increasing IL-10. In addition, the expression of IL-6, F4/80, CD11c, and TNFα was weakened. The results obtained were compared to the results obtained in the control group, in which the HF diet or a low-fat (10% fat) diet was used [53].

### 6.4. Anticancer Effect

In an experiment conducted by Nazeri et al. [54], it was confirmed that the ethanol extract of *B. oleracea* var. *acephala* reduced the viability of PC3 prostate cancer cells using the MTT test. PC3 cells treated with the tested extract were characterized by a decrease in the level of BCL-2 and NF-κB pathway genes and an increase in the level of BAX and NRF2 pathway genes, as well as an increase in mRNA levels. On the other hand, apoptosis was induced by a decrease in MMP, an increase in cytochrome c release, and activation of DNA fragmentation [54].

Gonçalves et al. [12] revealed in their research that the hydroalcoholic extract from the leaves of *B. oleracea* var. *acephala* does not have genotoxic and clastogenic activity but has antigenotoxic properties. The study was conducted on white mice. The first experimental group was a control and a positive control, which received only doxorubicin (DXR) at a dose of 80 mg/kg. The next group was administered 500, 1000, and 2000 mg/kg of the extract from the leaves of *B. oleracea* var. *acephala* in monotherapy by gavage. The last group received the extract from the leaves of *B. oleracea* var. *acephala* at the same doses together with DXR at a dose of 80 mg/kg. The analysis was performed in bone marrow cells using the micronucleus test and also in leukocytes, testicular, brain, bone marrow, and liver cells using the comet test. The hydroalcoholic extract of the leaves of *B. oleracea* var. *acephala* was shown to inhibit DXR-induced DNA damage at these doses. The researchers suggest that these properties may be beneficial in cancer prevention [12].

Another study conducted by Fagundes confirmed the antigenotoxic effect of squeezed juice from *B. oleracea* var. *acephala* leaves and also confirmed the clear lack of clastogenic and genotoxic effects. The experiment was conducted on male mice, which were orally administered 0.1 mL/10 g body weight of commercial or natural juice from *B. oleracea* var. *acephala* leaves, 2.5 mg/kg body weight of beta-carotene, 0.2 mg/kg body weight of lutein, and water. The positive control group received 25 mg/kg body weight of cyclophosphamide or 40 mg/kg body weight of methyl methanesulfonate to induce mutations and DNA damage. The experiment showed that the administered juice from *B. oleracea* var. *acephala* leaves caused a significant decrease in mutations and DNA damage in the group that received juice without added carotenoids. Researchers indicate that the demonstrated properties may have high therapeutic importance [55].

### 6.5. Antihypercholesterolemic Activity

In the study by Kim et al. [56], the aim of which was to determine the effect of kale juice consumption on reducing the risk factors of coronary heart disease in a group of men suffering from hypercholesterolemia. The subjects consumed 150 mL of fresh kale juice (equivalent to 167 g of fresh kale) daily for 12 weeks. It was found that the level of LDL cholesterol in the serum was significantly reduced by 10%, while the level of HDL cholesterol increased significantly by 27%. The ratio of HDL cholesterol to LDL cholesterol was also significantly increased by 52%. The researchers suggest that the consumption of kale juice has a beneficial effect on the lipid profile in the serum of the group of men studied, which leads to a lower level of risk of coronary heart disease in this group [56].

The study by Han et al. [57] was conducted in men with subclinical hypertension and focused on the relationship between their genetic polymorphisms of GST. It was shown that daily consumption of 300 mL of 100% pure kale juice for 6 weeks contributed to a decrease in plasma LDL cholesterol concentration, while it increased HDL cholesterol concentration in the case of the GSTT-present genotype [57].

### 6.6. Lowering Blood Sugar Level

A blinded, randomized, controlled trial lasting 12 weeks conducted by Jeppesen et al. [58] showed that eating bars containing 78 g of freeze-dried kale per day (equivalent to 341 g of fresh kale) by people with type 2 diabetes had a positive effect on fasting blood glucose levels. The control group received bars without added kale. A significant decrease of −1.4 in fasting glycated hemoglobin HbA1c was observed in the study group from the beginning to the end of this experiment compared to the control group. In this group, there was an increase of 1.4 in this parameter [58].

In another study conducted by Kondo et al. [59], consumption of 7 g or 14 g per day of kale has been shown to have significant inhibitory activity on postprandial plasma glucose levels. In patients taking 14 g of kale, the maximum plasma glucose concentration (Cmax) was 1.62 g/L after a meal. In the lower dose of 7 g, the Cmax value was slightly different, being 1.63 g/L [59].

According to Han et al. [57], in individuals with the GSTM1-present and GSTT1-null genotype polymorphism, significantly reduced blood glucose levels were observed after daily supplementation with 300 mL of 100% kale juice for 6 weeks [57].

### 6.7. Effects on Intestinal Microflora

In the study conducted by Shahinozzaman et al. [53], supplementation of an HF diet with 9% kale showed an impact on the composition and metabolic function of the intestinal microbiota in mice. A reduced ratio of *Firmicutes* to *Bacteroidetes* was noted. In addition, this diet caused the most abundant increase of about four-fold within the *Coriobacteriaceae* family and more than two-fold of the species *Bacteroides thetaiotaomicron* compared to the control group, which was given an HF diet without kale supplementation [53].

A randomized controlled trial conducted by Nishimoto et al. [60] documented that eating kale improves bowel movements and modifies some gut microorganisms. The study group consumed 7 g of powdered kale leaves mixed with 100–150 mL of water, and the control group consumed maltodextrin powder and cornstarch with the same amount of water. The prepared mixture was given to both groups twice a day for 4 weeks. The frequency of bowel movements increased in the experimental group, especially in patients with a smaller amount of stool before eating kale. There was a decrease in the number of microflora in the *Ruminococcus gnavus* strain, while there was an increase in *Eubacterium eligens* [60].

### 6.8. Summary of Biological Activities of Kale Based on Experimental Studies

Table 6 provides a comprehensive summary of the biological activities of *B. oleracea* var. *acephala* (kale), consolidating key findings from both in vitro and in vivo studies. It highlights the diverse health-promoting effects of kale, including antioxidant, antimicrobial, anti-inflammatory, anticancer, antihypercholesterolemic, and glycemic-regulating properties, as well as its impact on gut microbiota.

## 7. Status as a Superfood

The nutritional value of products is one of the key pieces of information for consumers, as well as indirectly indicating the nutrient density often considered in the context of superfoods. Literature data for kale, as for many other leafy vegetables, is relatively difficult due to its low proportion of dry matter. In the available literature as well as in databases, information can be found for both fresh leaves and dried plants. The approximate nutritional value of kale is shown in Table 7.

The term superfood is used both in scientific nomenclature and as part of the marketing of foodstuffs. However, it should be noted that it has not yet been fully defined either in the literature or in legislation [64]. It is also noteworthy that the term does not function in the records of major organizations such as the Food and Agriculture Organization (FAO), the World Health Organization (WHO), or the European Food Safety Authority (EFSA), although in the case of the latter, the concept of health claims has been introduced, which is applied to nutrient-dense foods. This definition coincides with the approach of many authors, who place kale in this category due to its nutrient richness [2,65,66].

The chemical composition of kale and its nutrient content is characterized by a rather high variability depending on the region of study or the material itself (fresh, freeze-dried) [30]. Noteworthy in this respect is the realistically high protein content and amino acid composition for plants of the *Brassicaceae* family. Analyzing dry matter, it was shown that its content decreases with the next harvest and ranges from 26.87% in the third harvest to 35% in the first harvest [31] on a dry matter basis. In fresh samples, the values are considerably lower, ranging from about 3 to almost 4%, depending on the report [11,36]. Nevertheless, a more important element in the assessment besides the protein content itself is its amino acid composition. Analyses of kale from this angle indicate a relatively high cysteine content (34–58 mg/100 g) [62,67], making kale a valuable source of this amino acid. Furthermore, in their study, Satheesh and Workneh Fanta [30] analyzed literature data on the amino acid composition of kale leaves, showing that these values are similar to those of other green leafy vegetables.

Similar data can also be found for other nutrients. Noteworthy in this respect are the minerals so far described for kale, or rather, the levels of their availability. Among vegetables grown in temperate climates, kale has the highest levels of potassium. The degree of bioavailability of the micronutrients deposited in kale is also an important feature. The degree of bioavailability of calcium from kale has been found to be maintained at a higher level than that of, for example, milk, which is customarily considered an excellent source of this micronutrient [68].

The nutrient composition of plant-based foods is significantly influenced by their culinary processing. For instance, in kale, steaming preserves 87–92% of phenolic compounds and antioxidant activity. In contrast, frying causes the most substantial nutrient degradation, with losses of 71% for chlorophylls, 28.2% for carotenoids, and 80–81% for flavanols. Ascorbic acid is particularly heat-sensitive, decreasing by 53.1% during boiling and 54.9% during frying. Frying also leads to the greatest reduction in overall antioxidant activity [69]. However, these data contradict reports by other authors, who indicate that the steaming process improves antioxidant capacity analyzed at the cellular and chemical levels, which may be related to easier extraction of these components from plant tissue [70].

Kale is also available in fixed, dried, or frozen forms. Research by Oliveira et al. [71] showed that drying contributes to significant losses of kale’s active ingredients. In contrast, a study by Vargas et al. [72] analyzing the content of glucosinolates and flavonoids in kale subjected to air-drying and freeze-drying, respectively, showed no significant differences, which may suggest that the reduction in bioactive components is directly related to the drying process itself and not its form. In the case of freezing, it was found that cell wall damage may facilitate the extraction of antioxidant-like compounds (increased antioxidant capacity) as well as phenolic compounds, while others, such as glucosinolates, remain relatively insensitive to changes associated with freezing fresh kale leaves [73].

Due to its low caloric value and high levels of bioactive compounds, kale has recently become one of the raw materials widely studied for its potential use not only as part of a balanced diet but also as a potential therapeutic ingredient in the development of some disease entities (Table 8).

As shown in Table 8, the active compounds in kale, through multidirectional regulation of the gut microbiome and modulation of metabolic and inflammatory pathways, have applications in improving gastrointestinal homeostasis and show therapeutic potential in the prevention of lifestyle diseases. Studies in a C57BL/6J mouse model with an obesity-inducing diet have shown that the bioactive components of kale significantly modulate the *Firmicutes*/*Bacteroidetes* ratio while increasing the diversity of the gut microbiome, resulting in a reduction in inflammatory markers [53]. This mechanism is associated with the induction of the formation of short-chain fatty acids (SCFAs), particularly butyrate, which plays an important role in maintaining the integrity of the intestinal barrier [74]. Further studies in models of intestinal inflammation have shown that kale exhibits anti-inflammatory effects by inhibiting the NF-κB pathway, leading to a decrease in pro-inflammatory cytokines such as TNF-α, IL-6, and IL-1β [52]. At the same time, an increase in Bacteroidetes was observed along with a decrease in *Enterobacteriaceae* populations, suggesting the prebiotic properties of kale. Importantly, compounds such as sulforaphane and flavonoids (kemferol, quercetin) not only strengthen the intestinal barrier by regulating the expression of tight junction proteins but also exhibit gastroprotective effects by reducing NSAID-induced gastric mucosal damage [75]. Reports of anticancer effects are particularly promising, as in vitro studies on colorectal cancer cell lines (HT-29, SW620) have shown that kale extracts induce apoptosis of cancer cells through activation of caspases (casp9) and the MAPK pathway (mapk10, mapk11) [76]. Additionally, fermented kale contains salicylic and gentisic acids, which have been shown to have antiproliferative properties [77]. In an in vivo model, kale-derived sulforaphane normalized the composition of the intestinal microbiota in mice with induced bladder cancer while inhibiting the NF-κB pathway and strengthening the intestinal barrier [78].

**Table 8:** Review of research on the status of kale as a superfood.

Category	Research Model	Key Findings	Mechanism of Activity	Research Methods	References
Regulation of the composition of the intestinal microbiome	C57BL/6J mice with diet-induced obesity	Feces modulate the composition of the intestinal microflora, reduce inflammation, and increase bacterial diversity.	Regulation of the Firmicutes/Bacteroidetes ratio, increasing the diversity of the microbiome.	16S rRNA sequencing, PICRUSt2 analysis	[53]
	C57BL/6J mice with intestinal inflammation	Kale protects against acute intestinal inflammation by modulating the ratio of pro- and anti-inflammatory bacteria and strengthening the intestinal barrier.	Increase in Bacteroidales, reduction in Enterobacteriaceae, and strengthening of the intestinal barrier.	Analysis of intestinal microflora, inflammatory markers	[52]
	Mice C57BL/6J	Consumption of kale affects the microbial ecology of the gut by increasing butyrate levels in the colon.	Increased production of short-chain fatty acids (SCFAs), especially butyrate.	HPLC for analysis of short-chain fatty acids	[79]
Anti-inflammatory effect	C57BL/6J mice with intestinal inflammation	Kale reduces inflammation in the gut by regulating pro-inflammatory cytokines and strengthening the intestinal barrier.	Inhibition of the NF-κB pathway, reduction in pro-inflammatory cytokines (TNF-α, IL-6, IL-1β).	Analysis of inflammatory markers, histopathological evaluation	[52]
	Wistar rats and Swiss mice	Kale shows gastroprotective effects by reducing gastric mucosal damage and lowering inflammatory markers.	Stimulation of gastric mucus secretion, reduction in gastric juice acidity through effects on pH and H^+^ concentration, protection against NSAID-induced damage through the modulation of prostaglandins, antioxidant and cytoprotective effects through flavonoids, and the presence of active compounds such as flavonoids (quercetin, kemferol), glucosinolates (e.g., sulforaphane), and terpenes and sterols.	Analysis of inflammatory markers, histopathological evaluation	[75]
Anticancer effects	Cancer cell lines (HT29, SW620)	Fermented kale contains salicylic and gentisic acids, which have been shown to have anticancer effects by reducing the number of cancer cells.	Induction of apoptosis, inhibition of tumor cell proliferation.	LC-MS, HPLC, immunoenzymatic analysis, histological evaluation of cells in a smear	[77]
	Mice with BBN-induced bladder cancer	Sulforaphane from kale normalizes intestinal microflora, strengthens the intestinal barrier, and reduces inflammation, which may protect against bladder cancer.	Regulation of intestinal microflora, strengthening of the intestinal barrier, and inhibition of the NF-κB pathway.	Analysis of intestinal microflora, inflammatory markers, and histopathological evaluation	[78]
	In vitro HT-29 cells	Kale extract reduced viability and inhibited proliferation of HT-29 cells.	elevated expression of casp9, mapk10, mapk11, fas, cat2 b, and ubd genes indicates apoptosis via the caspase-dependent pathway.	MTT and LDH assays; qRT-PCR	[76]

## 8. Status as a Cosmetic Ingredient

Kale is rich in bioactive compounds, including carotenoids (lutein, β-carotene), flavonoids, and vitamins (C, K), and presents promising applications in cosmetic formulations for both oral supplementation and topical administration. When administered systemically, its active constituents demonstrate antioxidant properties and stimulate collagen synthesis by scavenging reactive oxygen species (ROS) and inhibiting matrix metalloproteinases (MMPs). Topical applications (e.g., as extract-enriched creams) provide direct photoprotection against oxidative stress while reinforcing epidermal barrier function. These complementary delivery routes exhibit synergistic anti-aging effects, positioning kale as a scientifically validated, multifunctional cosmetic ingredient with clinically relevant dermatological benefits.

Given the properties of kale, the fact that its elements are valuable cosmetic raw materials seems quite significant. According to the Cosmetic Ingredients (CosIng) database [79], kale is incorporated into cosmetic formulations as a leaf extract with humectant and skin-conditioning functions, a seed oil acting as a skin-protecting agent, a plant powder obtained from the dried, ground plant used for skin conditioning, and a hydrolyzed protein blend (kale, carrot, and lemon) employed for hair conditioning and miscellaneous skin-conditioning purposes (Table 9).

Table 10 shows the literature evidence for the positive effects of various forms of kale as a bioactive ingredient in cosmetics. Studies using animal models seem to support the claim that kale extracts can have an inhibitory effect on skin aging. Studies in the SAMP1 mouse model showed that supplementation with glucoraphanin-enriched kale (GEK) significantly slows these processes through a multidirectional effect: activation of the Nrf2 pathway (increasing HO-1 and NQO1 expression), inhibition of Smad7 (which releases the TGF-β/Smad3 pathway and stimulates type I collagen synthesis), and improvement of ECM architecture (increasing elastin and hyaluronic acid levels). Importantly, these effects are dose-dependent, and the mechanism of action includes both reduction in oxidative damage and direct stimulation of repair processes [80]. It should be noted that in multi-component formulations, such as those combining kale with apple, green tea, or amino acids, the observed effects cannot be attributed solely to kale. Rather, they reflect the synergistic activity of all ingredients, with kale contributing through its antioxidant and skin-conditioning properties (Table 10).

A relatively new strand of research into the use of bioactive ingredients in cosmetology is the use of exosome-like nanobubbles, which allow for relatively precise delivery of bioactive ingredients to their target sites due to full control of the manufacturing processes. Studies on exosome-like nanobubbles derived from glucoraphanin-enriched kale have demonstrated their ability to modulate the TGF-β/Smad pathway in human skin fibroblasts. The key mechanism is the inhibition of Smad7 protein expression by microRNAs contained in the exosome-like nanobubbles, leading to activation of the TGF-β pathway and increased synthesis of extracellular matrix components such as type I collagen, elastin, and hyaluronic acid. Interestingly, this effect is dose-dependent, suggesting that the process can be precisely controlled [81].

On the other hand, studies conducted by Kappler et al. [82] on human epidermal fibroblast cell lines using a commercial cosmetic preparation of fenugreek and kale have shown that the use of these extracts not only has a positive effect on skin properties such as elasticity and hydration but also improves barrier function. The authors found that, due to their high antioxidant content, the mixture of these extracts protected the cells from oxidative stress caused by airborne pollutants or induced by H_2_O_2_. Thanks to the antioxidant properties of the preparation, the level of protein carbonylation and the effect of protein oxidation remained significantly lower than in the control group.

The role of antioxidant compounds present in kale in improving skin condition has also been demonstrated in studies conducted by Meinke et al. [83]. The research showed that supplementation with a carotenoid-rich kale extract (1650 µg/day) for 10 months significantly increased skin carotenoid levels and improved the SAAID index, reflecting the type I collagen-to-elastin ratio, indicating protection against collagen fiber degradation and maintenance of proper extracellular matrix structure. The study confirmed that carotenoids in kale exert antioxidant effects by neutralizing UV-induced free radicals, thereby inhibiting photoaging processes and improving skin quality, particularly in sun-exposed areas such as the cheeks. The results demonstrate that long-term supplementation with kale-derived carotenoids may effectively support a youthful skin appearance by preserving collagen and modulating dermal matrix structure.

Due to its high amount of bioactive components, kale, in its various forms, can be used not only as a preventive element in skin lesions but also as a therapeutic element. In a study by Zhang et al. [84], curly kale extract in combination with apple and green tea extracts and L-arginine was shown to have significant therapeutic effects in atopic dermatitis.

The study was conducted in two stages. Using an in vitro model of human keratinocytes (NHEKs) stimulated with cytokines (IL-22, TNF-α, IL-13, IL-4), a reduction in the expression of key inflammatory mediators (IL-24, CCL26) and stimulation of the synthesis of proteins essential for normal epidermal barrier function, including filaggrin, loricrin, and type I collagen (COL1A1), were observed. These results were associated with potent antioxidant properties (confirmed by an RPF value of 690 × 10^14^ radicals/mg), as well as modulation of inflammatory pathways associated with the Th2 response. In a 4-week, double-blind, placebo-controlled clinical trial involving patients with mild-to-moderate AD, therapy with a cream containing the aforementioned mixture of kale-apple and green tea extracts produced significant therapeutic benefits. Compared to placebo, a 63.5 percent reduction in the local SCORAD index (vs 22.6 percent in the control group), a significant reduction in pruritus, and an improvement in epidermal barrier function, as measured by a reduction in transepidermal water loss (TEWL), were observed. This effect is probably due to the synergistic action of the active ingredients, including the carotenoids and flavonoids present in kale, which not only neutralize reactive oxygen species but also stimulate the restoration of the epidermal structure.

## 9. Plant Biotechnology Studies

One of the several branches of plant biotechnology is genetic transformation, which holds great potential for improving the genetic traits of plants from the *Brassica* genus. This can bring significant agricultural and economic benefits. Meanwhile, micropropagation not only supports the regeneration process of plants after transformation but also plays a crucial role in maintaining genetic stability [85]. In 1988, an attempt was made to create interspecific hybrids between *B. oleracea* L. and *Brassica napus* L. The study used the embryo culture technique to develop hybrids. Currently, a more popular method is in vitro embryogenesis using culture of microspores [86]. The plant biotechnology studies on *B. oleracea* var. *acephala* are concentrated on micropropagation as well as on genetic transformation.

Micropropagation of species is the most widely studied issue. Depending on the approach, the explant can be hypocotyl, cotyledon, apical meristems, protoplasts, or callus, as well as microspores. An experiment was conducted to determine the effect of temperature on the development of somatic embryos from microspores of *B. oleracea* var. *acephala*. Microspores were subjected to heat shock at 32 °C and 35 °C for 2 days. It turned out that the optimal temperature for the embryogenesis of microspores of *B. oleracea* var. *acephala* was 35 °C. On the other hand, at a temperature of 32 °C, embryo stimulation turned out to be more effective [87].

Another study assessed the effect of amiprophosmethyl on the induction of polyploidy in a hybrid *B. oleracea* var. *acephala* and *Raphanus sativus*, and then the effect of aminoethoxyvinylglycine (AVG) on plant survival. It was shown that AVG increased the survival and production of tetraploids, which were characterized by larger shoot and leaf size compared to hexaploids and octaploids. In tetraploids, the level of glucosinolates was maintained, while in hexaploids and octaploids it decreased [88].

In the experiment, shoots were regenerated in vitro in *B. oleracea* var. *acephala* Alef from cotyledon and hypocotyl explants. Optimal regeneration was obtained on Murashige and Skoog (MS) medium with the addition of 0.1 mg/L of naphthylacetic acid (NAA) and 3 mg/L of 6-benzylaminopurine (BAP). A higher regeneration frequency of 76% was demonstrated by hypocotyl explants compared to cotyledons, which was 65%. The number of shoots per explant was 8.2 for hypocotyls and 4.3 for cotyledons. Moreover, the addition of 3 mg/L AgNO_3_ to the medium was shown to have a positive effect on shoot regeneration [89].

Another study investigated the effect of 6-furfuryladenine, 6-benzyladenine, and thidiazuron (TDZ) at various concentrations added along with NAA at lower concentrations on regeneration from hypocotyls of *B. oleracea* var. *acephala*. Histological examination confirmed two regeneration mechanisms: shoot organogenesis and somatic embryogenesis. Both pathways occurred simultaneously in the same explants, regardless of the combination of NAA and cytokines. The highest regeneration frequency of 95.9%, together with 7.5 morphogenetic structures per explant, was obtained with the combination of 2 µM NAA with 20 µM TDZ. High proliferation of somatic embryos occurred with the addition of 5 µM TDZ, generating 7.79 secondary embryos with the least degree of embryo abnormality. Moreover, plants grown on substrates containing TDZ had a higher phenol content [90].

A study was conducted to investigate the effects of BAP, 2-isopentenyladenine (2iP), and kinetin (Kin) added to the composition of MS medium, sterol metabolism, and cell membrane integrity of *B. oleracea* convar. *acephala* var. *sabellica* in vitro. It was shown that 2iP and BAP significantly stimulated shoot growth but weakened the efficiency of photosynthesis due to the reduced content of chlorophylls and carotenoids. On the other hand, all cytokines increased the respiratory rate, which indicated intensive metabolism. Moreover, they stimulated the synthesis of sterols, especially sitosterol, which is crucial for membrane stability and cell proliferation. 2iP and BAP increased the activities of lipoxygenase and phospholipase D, which resulted in a significant increase in membrane permeability [91].

Another study attempted to determine the culture conditions ensuring the highest efficiency of micropropagation of *B. oleracea* convar. *acephala* var. *sabellica*. The explant was the tops of kale shoots. The most efficient multiplication of shoots was achieved by adding BAP to the MS medium at a concentration of 2.5 mg/L. The most effective rooting was obtained by adding 1 mg/L of indolyl-3-acetic acid to the MS medium, where 95% of the shoots developed roots. In turn, the highest level of survival during the acclimatization process to ex vitro conditions was achieved by planting plants in a mixture of soil and perlite [92].

The researchers conducted an experiment to optimize the protocol for plant regeneration from protoplasts of selected *B. oleracea* varieties, including *B. oleracea* var. *sabellica* and *B. oleracea* var. *viridis*. The protoplasts were embedded in alginate layers and then cultured in three different types of media. The survival rate of protoplasts in all analyzed varieties was 88.2%. They underwent the process of cell wall resynthesis while they entered mitotic divisions on the fifth day of culture. After 30 days, the development of cell colonies in microcallus, derived from the protoplasts, was observed. The highest shoot formation efficiency of the tested species, including *B. oleracea* var. *sabellica* and *B. oleracea* var. *viridis*, was demonstrated in medium supplemented with 1 mg/L 2iP, 1 mg/L NAA, 0.02 mg/L GA_3_, and 2% mannitol [93].

The influence of the composition of the culture medium and cold pre-incubation on the embryogenesis of microspores and subsequent embryo regeneration of *B. oleracea* var. *acephala* was investigated. High efficiency of microspore embryogenesis was documented using liquid NLN medium with 16% sucrose without medium exchange during the culture. It was found that medium exchange significantly reduced the number of embryos obtained in all three genotypes tested: ‘Peachy Dancing’, ‘Nagoya’, and ‘P3’ compared to continuous microspore culture in the original medium. Among the genotypes tested, the highest number of embryos was obtained in the ‘Peachy Dancing’ cultivar, reaching an average of 123.63 embryos per dish. The application of 48 h cold pre-incubation of flower buds before microspore isolation in the cultivars ‘P3’ and ‘Nagoya’ significantly increased the efficiency of subsequent microspore embryogenesis. In the case of the cultivar ‘Peachy Dancing’, such treatment had a negative effect on this process. Cotyledon embryos were incubated on basic B5 medium, while abnormal embryos were incubated on B5 medium with the addition of 2% sucrose, 1.5 mg/L BAP, and 0.25 mg/L NAA. In both cases, high-quality embryos were obtained, from which healthy and strong plants grew. Among the genotypes studied, the percentage of spontaneously diploid plants derived from microspores ranged from 38% to 50% [94].

In another study, microspores were isolated from three varieties of *B. oleracea* var. *acephala* and treated with suberoylanilide hydroxamic acid to induce embryogenesis. It was shown that for Crane Feather Queen the optimal concentration of suberoylanilide hydroxamic acid was 0.03 μM. 17.27 embryos per bud were obtained. In turn, for Crane Bicolor and Crane Pink varieties, the appropriate concentration of suberoylanilide hydroxamic acid was 0.045 μM. This allowed obtaining 6.10 and 11.19 embryos per bud, respectively. In addition, haploid seedlings of microspores of Crane Feather Queen were doubled by two methods using colchicine. They were treated for 7 days with a colchicine solution at a concentration of 75 mg/L. The doubling index was 41.7%. The second method consisted of soaking the seedling roots for four hours and treating them with a 1000 mg/L colchicine solution. Then the doubling index was 64.3% [95].

The aim of another study was to evaluate the effect of sodium L-ascorbic acid (VcNa) on microspore embryogenesis and to evaluate the efficacy of colchicine in doubling the chromosome number in four genotypes of *Brassica oleracea* var. *acephala*. NLN-13 medium was supplemented with five concentrations of VcNA (1–5 μM). The highest frequency of embryogenesis was demonstrated for the BS-B genotype on the medium supplemented with 4 μM VcNA. It was 12.92-fold higher compared to the control, which was not supplemented with VcNA. However, at a concentration of 5 μM VcNA, the frequency of microspore embryogenesis decreased in all genotypic strains. The highest efficiency of doubling the number of chromosomes was achieved by immersing the roots in a colchicine solution at concentrations of 750 mg/L for 4 to 6 h and 1000 mg/L for 2 h. This led to doubling the chromosomes in almost 50% of haploids [96].

In another study, the efficiency of *B. oleracea* var. *acephala* embryogenesis was investigated by adding BAP, NAA, activated carbon, *p*-chlorophenoxyisobutyric acid, and arabinogalactan to the medium. Promising results were obtained by adding a combination of NAA (0.1–0.2 mg/L) and BAP (0.1–0.2 mg/L) or activated carbon (0.1–0.2 g/L) and arabinogalactan (0.1–0.2 g/L). The efficiency of embryogenesis was increased by adding 40 μM *p*-chlorophenoxyisobutyric acid or a combination of 40 μM *p*-chlorophenoxyisobutyric acid and 0.2 g/L activated carbon to the NLN-13 medium. The highest embryonic induction rate was demonstrated during treatment with the combination of *p*-chlorophenoxyisobutyric acid and 10 mg/L arabinogalactan [97].

Microspores were isolated from three cultivars (Crane Feather Queen, Crane Pink, and Crane Bicolor) of the cut flower *B. oleracea* var. *acephala* and treated with trichostatin A to induce embryogenesis. It was proven that the optimal concentration of trichostatin A for Crane Bicolor was 10 nM. Then, 5.39 embryos per bud were obtained, while embryo mortality decreased to 25.01%. On the other hand, the corresponding concentration of trichostatin A for the cultivars Crane Feather Queen and Crane Pink was 5 nM. Then, 16.99 and 10.89 embryos per bud were obtained, respectively, and embryo mortality decreased to 30.03% and 29.32% [98].

The aim of another experiment was to determine the effect of adding methylene blue (MB) to the medium on the process of microspore embryogenesis and regeneration for four genotypes of *B. oleracea* var. *acephala*. It was shown that a favorable rate of embryogenesis stimulation occurred at a concentration of 8 nM MB, but it was most optimal at a concentration of 10 nM MB [99].

Some of the first studies on genetic transformation of kale concerned obtaining transgenic plants through infection with *Agrobacterium tumefaciens* and *A. rhisogenes* [98,99,100]. One of them focused on the genetic transformation of *B. oleracea* var. *acephala*, which was performed using an *Agrobacterium rhizogenes* vector system. Leaf petioles of *B. oleracea* var. *acephala* were inoculated with an *A. rhizogenes* suspension containing the pBI 121 plasmid. From two roots that gave a positive X-gluc reaction result, five shoots were regenerated on MS medium with the addition of carbenicillin sodium and growth regulators. As a result, the shoots survived, which confirms that the transformation was effective [100].

Another application of *Agrobacterium* transformation is the production of hairy root cultures. Due to their specificity and growth rate, these cultures can be a rich source of metabolites. Korean researchers conducted a comparative analysis of GSL biosynthesis in the hairy roots of green and red kale (*Brassica oleracea* var. *acephala*), induced using *Agrobacterium rhizogenes* R1000. The aim was to determine differences in the expression of genes involved in the GSL biosynthesis pathway and the accumulation of specific secondary metabolites in both varieties. It was shown that most genes associated with indolic GSL biosynthesis exhibited higher expression in the hairy roots of green kale. In contrast, the expression of key enzymes in the aromatic GSL biosynthesis pathway, encoded by *BoCYP83A1* and *BoSUR1*, was significantly higher in red kale. The concentrations of 4-hydroxyglucobrassicin, glucobrassicin, and 4-methoxyglucobrassicin were 2-, 5.98-, and 6.21-fold higher, respectively, in green kale compared to red kale. On the other hand, the content of gluconasturtiin and neoglucobrassicin was significantly higher in red kale, 3.48- and 16.2-fold, respectively, compared to green kale [101].

## 10. Conclusions

Kale (*Brassica oleracea* var. *acephala*) emerges as a multifunctional plant species with exceptional nutritional, therapeutic, and functional food potential. This integrative review highlights its rich phytochemical profile, including glucosinolates, polyphenols, carotenoids, flavonoids, and essential vitamins and minerals, which collectively contribute to its classification as a “superfood”. The bioactive compounds derived from kale, particularly sulforaphane, indole-3-carbinol, and diindolylmethane, exhibit potent antioxidant, anti-inflammatory, antimicrobial, and anticancer properties, substantiated by both in vitro and in vivo studies.

Clinical and preclinical evidence supports the role of kale in mitigating risk factors associated with chronic diseases such as cardiovascular disorders, type 2 diabetes, and hormone-related cancers. Furthermore, kale demonstrates beneficial effects on gut microbiota modulation, glycemic control, lipid metabolism, and systemic inflammation, reinforcing its value in preventive nutrition and integrative medicine.

From a regulatory perspective, kale is recognized as a safe and beneficial dietary component by major health authorities. The U.S. Food and Drug Administration (FDA) includes kale among those generally recognized as safe [29]. The EFSA acknowledges its nutritional value and bioactive potential in the context of health claims and dietary recommendations [66]. Similarly, the European Medicines Agency (EMA) considers kale relevant in the context of traditional herbal medicinal products [102]. Moreover, kale is listed in the European Commission’s CosIng database as an approved cosmetic ingredient, where it is valued for its antioxidant and skin-conditioning properties [79].

Advances in plant biotechnology, including micropropagation, somatic embryogenesis, and genetic transformation, offer promising avenues for enhancing the bioactive potential and agronomic traits of kale. These innovations not only support sustainable cultivation but also facilitate the development of novel functional food products, nutraceuticals, and cosmeceuticals.

In conclusion, *B. oleracea* var. *acephala* represents a highly promising candidate for health-promoting dietary strategies, biotechnological innovation, and cosmetic applications. Its integration into modern diets, therapeutic protocols, and skincare formulations may contribute significantly to public health improvement and disease prevention. Future research should focus on clinical validation of its health benefits, optimization of bioactive compound extraction, and exploration of its potential in personalized nutrition and functional product development.

From a scientific perspective, this review fills a gap by integrating the most recent data on kale’s phytochemistry, biological activity, and technological applications. It not only consolidates current knowledge but also identifies emerging trends and research directions, particularly in the fields of plant biotechnology and cosmeceutical development. As such, it serves as a valuable reference for researchers, clinicians, and product developers seeking to explore the full potential of *B. oleracea* var. *acephala* in health-related and industrial contexts.

## Figures and Tables

**Table 1:** *Brassica oleracea* var. *acephala* cultivar varieties.

Variety	Trade Name Examples	Morphological Appearance
Curly (green)	Darkibor Dwarf Green Curled Afro Pentland Brig Meadowlark Ripbor Winterbor Vates Blue Ridge Blue Knight Maribor	long central stems and distinctly twisted or wavy, dark green leaves
Curly (red)	Baltic Red Redbor Roulette Scarlet	strongly curly leaves in shades of pink, red, and purple
Portuguese	Beira	wide, mostly whole leaves
Russo-Siberian	Dwarf Siberian Gulag Siberian True Siberian Fizz	flatter, wavy, green leaves (Red Russian has dark red stems and leaf veins)
Collard	Top Bunch Champion Georgia Collard Green Glaze Evenstar	smooth, wide, dark green leaves
Dinosaur	Black Magic Lacinato	dark green leaves with a rough texture

**Table 2:** Average nutrient content in 100 g of fresh *Brassica oleracea* var. *acephala* and Reference Daily Intakes (RDIs) according to the USDA.

Component			Content per 100 g of Fresh Weight	RDI
water			89.6 g	2.7–3.7 L/d
protein			2.92 g	50 g
fat			1.49 g	78 g
carbohydrates			4.4 g	275 g
fiber			4.1 g	28 g
MINERALS	Macroelements	potassium (K)	348 mg	4700 mg
		calcium (Ca)	254 mg	1300 mg
		sodium (Na)	53 mg	2300 mg
		magnesium (Mg)	32.7 mg	420 mg
	Microelements	iron (Fe)	1.6 mg	18 mg
		phosphorus (P)	55 mg	1250 mg
		zinc (Zn)	0.39 mg	11 mg
		manganese (Mn)	0.92 mg	2.3 mg
		copper (Cu)	0.053 mg	0.9 mg
VITAMINS		vitamin A	241 μg	900 μg RAE *
		lutein and zeaxanthin	6260 μg	nd **
		thiamine (vit. B_1_)	0.113 mg	1.2 mg
		riboflavin (vit. B_2_)	0.347 mg	1.3 mg
		niacin (vit. B_3_)	1.18 mg	16 mg
		pantothenic acid (vit. B_5_)	0.37 mg	5 mg
		pyridoxine (vit. B_6_)	0.147 mg	1.7 mg
		acid folic (folate, vit. B_9_)	62 μg	400 μg DFE ***
		vitamin C	93.4 mg	90 mg
		vitamin E	0.66 mg	15 mg
		vitamin K	390 μg	120 μg

* RAE = Retinol Activity Equivalents; 1 microgram RAE = 1 microgram retinol, 2 microgram supplemental β-carotene, 12 micrograms β-carotene, 24 micrograms α-carotene, or 24 micrograms β-cryptoxanthin. ** nd = No data. *** DFE = Dietary Folate Equivalents; 1 DFE = 1 mcg naturally occurring folate = 0.6 mcg folic acid.

**Table 3:** Isothiocyanates occurring in *Brassica oleracea* var. *acephala*.

Glucosinolate Name	Isothiocyanates
Glucoerucin	4-methylthiobutyl glucosinolate
Glucoiberin	3-methylsulfinylpropyl glucosinolate
Glucoraphanin	sulforaphane, 4-methylsulfinylbutyl glucosinolate
Gluconasturcin	2-phenylethyl glucosinolate
Glucobrassicin	3-indolylmethyl glucosinolate
Progoitrin	2-hydroxy-3-butenyl glucosinolate

**Table 4:** Health-promoting effects of glucosinolate’s metabolites found in *Brassica oleracea* var. *acephala*.

Compound	Action	References
Indole-3-carbinol	chemoprevention by inhibiting DNA adduct formation and cell proliferation, inhibiting the cell cycle and invasive growth along with angiogenesis, and inducing apoptosis	[33]
	induction of enzymes of phase I and II of detoxification	[39]
	prevention of cervical, endometrial, and breast cancer	[41]
	hydroxyestrone formation and regression of cervical intraepithelial neoplasia	[41]
Diindolylmethane	DNA repair	[44]
	activation of the estrogen receptor α (ERα) signaling pathway	[45]
Sulforaphane	protection of nerve cells in Parkinson’s disease	[43]
	bactericidal vs. *Helicobacter pylori*	[42]
	↓ concentrations of active carcinogens	[24]
	↓ leptin and cholesterol levels ↑ adiponectin concentrations	[7]

**Table 5:** Active compounds and related health-promoting activity of *Brassica oleracea* var. *acephala* leaves.

Group of Compounds	Active Compound	Biological Activity Related to Plant Consumption	References
Phenolic acids	sinapic acid	NF-κB inhibition, antiproliferative effect on breast cancer cell lines	[7]
	ferulic acid	antioxidant, anti-inflammatory, antidiabetic, antihypertensive, antimicrobial, antiviral	[7]
Flavonoids	quercetin, kaempferol	antioxidant, anti-inflammatory, antimutagenic, anti-edematous, anti-allergic, anti-atherosclerotic, and gastroprotective	[12,30]
		neuroprotective, normotensive, vasodilatory, antiproliferative	[7]
Carotenoids	β-carotene and lutein	antioxidant, skin protection against UV rays, stimulation of the immune system, regulation of the cell cycle and growth factors, modulation of intercellular signaling pathways,	[7,48]
		protection against cardiovascular disease, cataracts, diabetes, prostate cancer, and cancers in the digestive tract	[7]
	lutein	antiproliferative, neuroprotective, antidiabetic, apoptosis-inducing effect	[7]
Chlorophylls	chlorophyll a and b	detoxification of the body	[47]
Vitamins	vitamin E	stabilization of cell membranes and prevention of oxidative damage to tissues	[2]

**Table 6:** Biological activities of kale.

Activity	Research Model	Key Findings	Mechanism of Activity/Results	References
In vitro studies				
Antioxidant potential	in vitro studies	The ethanolic flower extract showed higher antioxidant potential than the ethanolic stem extract.	The flower extract showed 146.56 µg AAE/mL compared to 80.35 µg AAE/mL from stems. DPPH inhibition was 80.80% for flowers and 13.25% for stems; activity increased with concentration.	[49]
	in vitro studies	Seasonal variation affected antioxidant activity in leaf extracts.	DPPH IC50 ranged from 1.91 to 1.41 mg/mL; FRAP values ranged from 29.77 to 13.43 µg AAE/g.	[50]
	in vitro studies	Methanol leaf extract showed antioxidant power.	The FRAP value was 29.35 µmol Fe^2+^/g DW.	[24]
	in vitro studies	Enzymatic antioxidant activity confirmed.	SOD, CAT, and POX enzymatic activities were IC50 = 30.06 mg; 38.6 mM H_2_O_2_/min/g of fresh mass; and 50.33 ΔA/min/g of fresh mass, respectively.	[23]
Antimicrobial activity	in vitro studies	Ethanol extract from flowers and stems showed antibacterial activity against several bacterial strains.	MIC values ranged from 0.625 mg/mL to 5 mg/mL against *Bacillus subtilis*, *Enterobacter aerogenes*, *Enterococcus faecalis*, *Gordonia rubripertincta*, *Klebsiella pneumoniae*, *Proteus vulgaris*, and *Salmonella enterica*.	[49]
	in vitro studies	Ethanol extract from the leaves showed antibacterial and antifungal effects.	Activity was observed against *Bacillus subtilis*, *Clostridium perfringens*, *Listeria monocytogenes*, *Staphylococcus aureus*, *Escherichia coli*, *Proteus vulgaris*, *Pseudomonas aeruginosa*, and *Salmonella enterica*. Activity was confirmed against *Candida albicans* and *Aspergillus niger*.	[51]
	in vitro studies	Ethanol extract from flowers and stems showed antifungal effects against several strains.	MIC values ranged from 0.625 to 1.25 mg/mL against *Candida albicans*, *Candida parapsilosis*, *Candida tropicalis*, and *Saccharomyces cerevisiae*.	[49]
Anticancer activity	in vivo (PC3 cells)	Ethanol extract reduced prostate cancer cell viability and induced apoptosis.	Natural juice lowered DNA damage even without added carotenoids; the protective effect was confirmed.	[12]
In vivo studies—animal model				
Anti-inflammatory activity	in vivo (mice)	An HF diet enriched with *B. oleracea* var. *acephala* reduced inflammatory gene expression in mice.	IL-6, IL-1β, TNFα, NF-κB, iNOS, and F4/80 expression levels were reduced compared to inflammation-induced controls.	[52]
	in vivo (mice)	Long-term kale supplementation suppressed pro-inflammatory markers and increased anti-inflammatory cytokines.	MCP-1 was reduced; IL-10 increased; and IL-6, F4/80, CD11c, and TNFα were downregulated.	[53]
Anticancer activity	in vivo (mice)	Hydroalcoholic leaf extract showed antigenotoxic effects without genotoxicity.	DNA damage induced by doxorubicin was inhibited in leukocytes, testicular, brain, liver, and bone cells.	[55]
	in vivo (mice)	Naturally squeezed juice has been confirmed to have a clear lack of clastogenic and genotoxic effects.	Natural juice lowered DNA damage even without added carotenoids; the protective effect was confirmed.	[54]
Effects on intestinal microflora	in vivo (mice)	Kale altered microbiota composition and function in HF diet-fed mice.	Reduced ratio of *Firmicutes* to *Bacteroidetes* and an increase in *Coriobacteriaceae* and *Bacteroides thetaiotaomicron*.	[60]
Antihypercholesterolemic activity	in vivo (humans)	Daily consumption of kale juice improved lipid profiles in hypercholesterolemic men.	LDL decreased by 10%, HDL increased by 27%, and the HDL/LDL ratio increased by 52%.	[56]
	in vivo (humans)	Genetic polymorphism influenced the effect of kale juice on cholesterol levels.	300 mL of juice lowered LDL and increased HDL in GSTT-present genotype individuals.	[57]
Blood sugar-lowering activity	in vivo (humans)	Kale bar consumption significantly lowered HbA1c in type 2 diabetes patients.	In the intervention group, there was a decrease of −1.4 in fasting glycated hemoglobin HbA1c compared to an increase in the control group.	[58]
	in vivo (humans)	Kale supplementation reduced postprandial glucose levels.	Cmax of glucose was 1.62 g/L for a 14 g dose and 1.63 g/L for a 7 g dose.	[59]
	in vivo (humans)	Genetic variation influenced glycemic response to kale juice.	Individuals with GSTM1-present and GSTT1-null genotypes showed significantly lower blood glucose levels.	[57]
Effects on intestinal microflora	in vivo (humans)	Kale improved bowel movement and modified flora composition.	Increased bowel movement; *Ruminococcus gnavus* decreased, and *Eubacterium eligens* increased in the experimental group.	[53]

**Table 7:** Nutritional value of kale.

Component	USDA Food Data Central [61] (Raw)	Ayaz et al. [62] (Dried Leaves)	Thavarajah et al. [63] *
Macronutrients			
Calories (kcal)	35 (2% DV)	nd	36–98
Protein (g)	2.9 (6% DV)	27.1 (54% DV)	1.6–5.9
Fat (g)	1.5 (2% DV)	0.158 (<1% DV)	nd
Carbohydrates (g)	4.4 (2% DV)	nd	nd
Fiber (g)	4.1 (15% DV)	nd	nd
Sugars (g)	0.99 (2% DV)	Fructose: 2.01, Glucose: 1.06	Glucose: 0.69–33.5, Fructose: 0.29–19.3, Sucrose: 0.004–2.12
Saturated fats (g)	0.18 (1% DV)	nd	nd
Vitamins			
Vitamin A, RAE (µg)	241 (27% DV)	nd	nd
Vitamin C (mg)	93.4 (104% DV)	nd	nd
Vitamin K (µg)	389.6 (325% DV)	nd	nd
Minerals			
Calcium, Ca (mg)	254 (20% DV)	1970 (197% DV)	35–300
Iron, Fe (mg)	1.6 (9% DV)	72.6 (405% DV)	0.5–2.3
Magnesium, Mg (mg)	33 (8% DV)	240 (60% DV)	20–100
Zinc, Zn (mg)	0.39 (4% DV)	39.4 (358% DV)	0.2–1.6
Manganese, Mn (mg)	0.92 (40% DV)	53.5 (2326% DV)	0.2–2.3
Copper, Cu (mg)	0.05 (6% DV)	5.1 (567% DV)	0.002–0.116
Potassium, K (mg)	348 (7% DV)	13,500 (288% DV)	188–873
Selenium, Se (µg)	0.9 (2% DV)	No data	0–17
Fatty Acids			
Omega-3 (ALA) (g)	0.378 (24% AI)	0.0853 (5% AI)	nd
Omega-6 (LA) (g)	0.291 (2% AI)	0.0186 (<1% AI)	nd
Amino acids (selected)			
Lysine (mg)	175 (8% RDI)	1500 (72% RDI)	nd
Tryptophan (mg)	35 (13% RDI)	890 (318% RDI)	nd

% DV (% Daily Value for a 2000 kcal diet); % AI (Adequate Intake—for fatty acids); % RDI (Reference Daily Intake for amino acids). * The result is given as a range—the authors analyzed the chemical composition of many varieties.

**Table 9:** Use of kale in cosmetic products based on data from the CosIng database [79].

Name	Description	Function
*Brassica oleracea acephala* leaf extract	*Brassica oleracea acephala* Leaf Extract is the extract of the leaves of *Brassica oleracea* L., var. *acephala*, *Brassicaceae*.	Humectant Skin Conditioning
*Brassica oleracea acephala* seed oil	*Brassica oleracea acephala* Seed Oil is the oil expressed from the seeds of *Brassica oleracea* L. var. *acephala*, *Brassicaceae*.	Skin Protecting
*Brassica oleracea acephala* Powder	*Brassica oleracea acephala* Powder is the powder obtained from the dried, ground plant, *Brassica oleracea* L., var. *acephala*, *Brassicaceae*.	Skin Conditioning
Hydrolyzed carrot protein/hydrolyzed kale protein/hydrolyzed lemon protein extract	Hydrolyzed Carrot Protein/Hydrolyzed Kale Protein/Hydrolyzed Lemon Protein Extract is the extract of the protein hydrolysates obtained from the roots of *Daucus carota sativa*, the leaves of *Brassica oleracea acephala*, and the pulp of *Citrus limon* derived by acid, enzyme, or another method of hydrolysis.	Hair Conditioning Skin Conditioning—Miscellaneous

**Table 10:** Chosen data on the potential use of kale in cosmetics.

Formulation	Model System	Key Findings	Mechanism of Action	Clinical Outcomes	References
Oral supplementation (glucoraphanin-enriched kale)	SAMP1 mouse model	Decelerated skin aging	Nrf2 activation (↑ HO-1, NQO1); Smad7 inhibition → TGF-β/Smad3 activation; ECM remodeling (↑ elastin, HA)	-	[80]
Exosome-like nanovesicles (kale-derived)	Human dermal fibroblasts	↑ ECM synthesis (collagen I, elastin, HA)	miRNA-mediated Smad7 suppression → TGF-β activation	Dose-dependent efficacy	[81]
Topical cosmetic (kale + fenugreek extract)	Human fibroblast cell lines	Improved elasticity/barrier function	Antioxidant protection (↓ protein carbonylation)	-	[82]
Oral supplement (1650 µg/day carotenoids)	10-month clinical trial	Skin matrix preservation	ROS scavenging; collagen/elastin ratio modulation (SAAID index)	↑ Skin carotenoids; improved photoaged skin	[83]
2% topical cream (kale + apple + green tea + L-arginine)	NHEKs (in vitro); AD patients (4-week trial)	Anti-inflammatory + barrier repair	Th2 modulation (↓ IL-24, CCL26); ↑ filaggrin/loricrin; RPF 690 × 10^14^ radicals/mg	63.5% ↓ SCORAD; ↓ pruritus/TEWL vs. placebo	[84]
Topical serum (5% kale extract)	Human keratinocytes (HaCaT)	Reduced MMP-1/3 (40–60%), increased collagen I synthesis	Nrf2/ARE activation; NF-κB inhibition (reduced TNF-α, IL-6)	Improved wrinkle depth (25%)	[82]

Abbreviations: ECM = Extracellular matrix; TEWL = Transepidermal water loss; NHEKs = Normal human epidermal keratinocytes; AD = Atopic dermatitis; RPF = Radical protection factor; SCORAD = Scoring Atopic Dermatitis index; HA = Hyaluronic acid.

## Data Availability

The data presented in this study are available on request from the corresponding author.
